# Facial Nerve Schwannoma of Parotid Gland: Difficulties in Diagnosis and Management

**DOI:** 10.1155/2016/3939685

**Published:** 2016-01-20

**Authors:** Murat Damar, Aykut Erdem Dinç, Sultan Şevik Eliçora, Sultan Bişkin, Gül Erten, Serdar Biz

**Affiliations:** ^1^Otolaryngology Department, Bülent Ecevit University Faculty of Medicine, 67600 Zonguldak, Turkey; ^2^Pathology Department, Bülent Ecevit University Faculty of Medicine, Zonguldak, Turkey

## Abstract

Facial nerve schwannomas (FNS) are encapsulated benign tumors arising from Schwann cells of seventh cranial nerve. Most of the facial nerve schwannomas are localized in intratemporal region; only 9% of cases involve a portion of the extratemporal segment. Preoperative diagnosis is often unclear; diagnosis is often made intraoperatively. Management of intraparotid FNS is troublesome because of the facial nerve paralysis. In this report we presented a case of intraparotid schwannoma in a 55-year-old male patient complaining of a painless mass without peripheral facial nerve palsy in left parotid gland. Clinical features, preoperative and intraoperative diagnosis, and difficulties during management are discussed with the review of the literature.

## 1. Introduction

Schwannomas (neurilemmomas) are rare benign, slow growing, encapsulated tumors that are arising from Schwann cells of myelinated peripheric or cranial nerves [1]. Twenty-five percent of all schwannomas originate from the neural structures of head and neck region. Facial nerve schwannomas (FNS) can arise from Schwann cells of seventh cranial nerve, at any part from the glial-Schwann cell junction at the cerebellopontine angle to the terminal branches of the medial face. Schwannomas of the facial nerve are uncommon. Most of the facial nerve schwannomas are localized in intratemporal region; only 9% of cases involve a portion of the extratemporal segment [1, 2].

Intraparotid FNS most commonly presents as a painless, slow growing parotid mass with normal facial nerve function [3]. Generally it is difficult to diagnose preoperatively. In this report we presented a case of intraparotid schwannoma in the left parotid region. Clinical features, preoperative and intraoperative diagnosis, and difficulties during management are discussed with the review of the literature.

## 2. Case Report

A 55-year-old male patient complaining of a painless mass in the left preauricular area that had been present for 3 months was admitted to our clinic. There was no other symptom and his general physical examinations were completely normal. Physical examination showed a 35 × 30 mm mass in left parotid gland that is partially mobile and painless and covered with normal skin. The facial nerve functions were normal on clinical evaluation. Fine-needle aspiration cytology (FNAC) result was nondiagnostic and complete blood count was normal.

**Figure 1:**
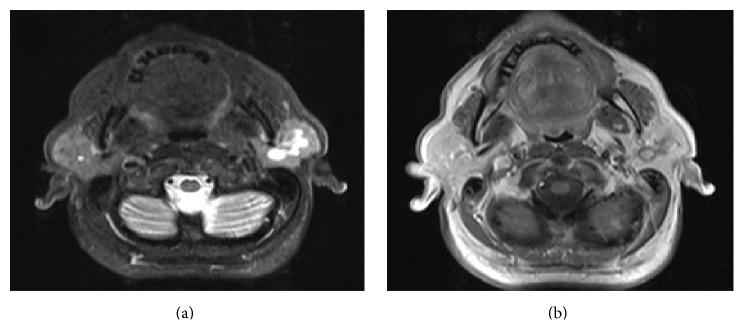
(a) In axial T2 FS magnetic resonance imaging (MRI) a homogeneous hyperintense lesion was seen in superficial and deep lobes of the left parotid gland. (b) In contrasted axial T1 sections a heterogeneous dense enhancement was seen in the center of the mass.

In magnetic resonance imaging (MRI) a homogeneous 30 × 20 mm lesion was detected in superficial and deep lobes of left parotid gland spreading to anterior of masseter muscle which is hypointense in T1A sections and hyperintense in T2A sections and with intense contrast enhancement (Figures 1(a) and 1(b)).

**Figure 2:**
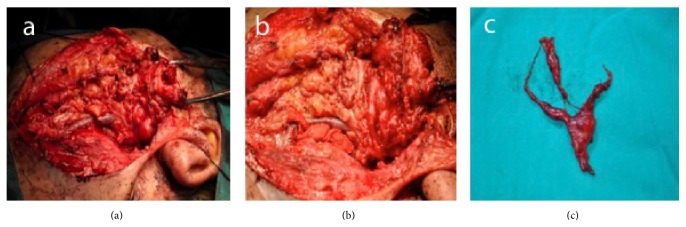
(a) The intraoperative view of the tumoral mass after explosion of the main trunk of facial nerve. (b) It is clearly seen after frozen section from the center of the mass that the tumor was surrounding the temporozygomatic branches of the facial nerve. (c) The postoperative view of the tumor after excision.

A parotidectomy was planned for the patient. During the operation firstly the main trunk of facial nerve was identified. The main part of the tumor was a 20 × 20 mm reddish, bright lesion with a thin capsule around the temporofacial branch of facial nerve anterosuperior to main trunk. The lesion was encircling the temporofacial branch of the facial nerve and advancing forward through the peripheral branches. At this point we could not follow the nerve, so we referred to frozen section. The result was reported as “mesenchymal lesion.” When we stimulated the lesion with the nerve stimulator we got an answer from the muscles that were innervated by that branch and because of the frozen section result we thought that the lesion might be a tumor of the nerve itself. In this occasion we cut the temporofacial branch of the nerve from the main trunk and excised the tumor totally with branches (zygomatic, temporal, and buccal branches) surrounding the mass medially (Figures 2(a), 2(b), and 2(c)). A weakness was detected in left eyelid postoperatively. The patient was able to close his eye with effort so no additional intervention was considered afterwards. The weak conservation of eyelid function despite the sacrificing of temporofacial branch could be explained by the compensation from collateral peripheral branches located in front of the resected tumor.

**Figure 3:**
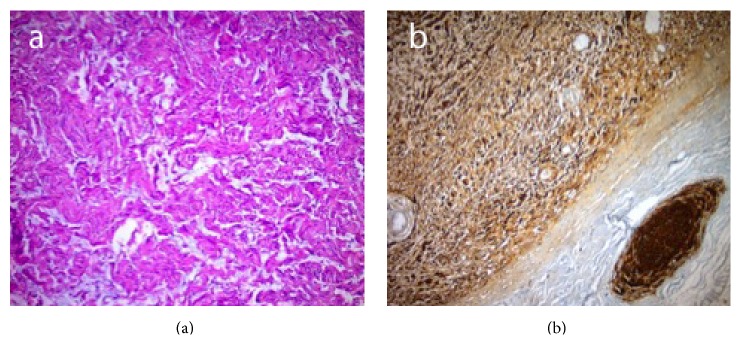
(a) Lesion was forming wavy fiber structures in edematous stroma in hypocellular areas. (b) Lesion had positive reaction with S-100 in immunohistochemical examination (a: Hematoxylin-Eosin X200, b: BSA-DAB X100).

The histopathologic result was reported as “schwannoma.” The cells that build the tumor were fusiform nucleated spindle cells and had eosinophilic cytoplasms with ill-defined boundaries. In these cells there was palisade forming hypercellular areas and wavy structure of the fibers in the stroma in some places. There was no necrosis, mitosis, or atypia. The neoplastic cells were vimentin and S-100 positive immunohistochemically (Figures 3(a) and 3(b)).

## 3. Discussion

Schwannomas were first reported by Virchow in 1908 and arise from the neural sheath of the peripheral sensory, motor, sympathetic, and cranial nerves [3, 4]. Schwannomas are encapsulated, soft and white, yellow, or pink tumors. They occasionally feature areas of calcification and/or cystic degeneration. Their capsule is continuous with the epineurium, the most external nerve sheath [5]. Microscopically the diagnosis is confirmed by histopathologic evaluation. Histologically, two types of tissues are seen: the Antoni A area is characterized by the presence of elongated and spindle-shaped Schwann's cells, and their nuclei are aligned in a palisading pattern (Verocay bodies). The hypo cellular Antoni B area has a varying degree of cell pleomorphism; irregular cell types are scattered in loose connective tissue, and there is no definable palisading of tumor cell nuclei. Both Antoni areas are usually found in the same tumor, but their respective proportions vary. Nerve fibers are not part of the tumor because the mass arises from Schwann's sheath and pushes the nerve axons aside [5, 6]. Immunostaining for S-100 is required to establish the neural origin of the tumor and smooth muscle actin (SMA) to rule out a leiomyoma [7].

FNS are uncommon; approximately 25% and 40% of all schwannomas have occurred in the head and neck region. These tumors are most common tumors of facial nerve and can be either benign or malignant. The majority of FNS are intratemporal, with 9% of cases arising from the intraparotid portion [8]. Caughey et al. had conducted a retrospective study over 38-year period, focusing on facial nerve schwannoma involving parotid gland. Out of a total of 3722 patients with schwannomas reviewed, only 29 were related to facial nerve. From this small group, only eight involved the parotid segment of the facial nerve [9].

Intraparotid FNS are solitary, painless, slow growing masses mimicking the tumors of the parotid gland and have a different clinical presentation. Although tumor arises from the nerve itself, the function of the facial nerve is generally unaffected [9, 10]. It is interfered with malignant parotid tumors if there are facial paralysis and pain. The incidence of intraparotid facial nerve palsy is approximately %20-27 [11]. The ability of the parotid gland to accommodate tumor expansion well and the propensity of tumor to grow eccentrically and push nerve fibers away account for the low rate of facial palsy in intraparotid FNS [12].

It is difficult to distinguish the intraparotid FNS from other parotid tumors preoperatively. But it is also essential to design the treatment. Computed tomography (CT) scan usually shows a smooth, sharply circumscribed lesion in the parotid. It is not a preferred imaging modality for FNS, but it may reveal osseous changes [13]. Sonographically, neurogenous tumors present as spindle-shaped masses with cystic areas [9]. MRI is the most appropriate imaging technic in detecting facial nerve schwannoma along the course of the facial nerve. It shows a mass with signal intensity isointense to muscle on T1 and hyperintense to muscle on T2 sequence, respectively [13]. The target sign (central low and peripheral higher signal intensity on T2 weighted images) is suggestive of a neurogenous tumor such as benign facial nerve schwannoma or malignant nerve sheath neoplasm [14]. FNAC is generally unsuccessful in diagnosis of intraparotid nerve schwannomas and results are inconclusive as in our case [7, 15, 16]. FNAC may reveal spindle-shaped cells with ill-defined cytoplasm, arranged in clusters (Verocay bodies) [17].

Treatment of intraparotid FNS is surgical excision. Because preoperative diagnosis is impossible, much responsibility falls to the surgeon during the operation. Because in almost all cases no facial nerve palsy is detected preoperatively; even during surgery, it can be difficult to determine whether the tumor originated from the parotid gland or the facial nerve; half of the tumors originate from the main trunk of the facial nerve and that is why it is impossible to find main trunk. There are strong adhesions between the tumor and the nerve and so in such cases surgery becomes quite difficult. During the operation if it is seen that tumor is adherent to the nerve and it is impossible to dissect them, intraoperative frozen section examination is recommended for both diagnosis and excluding malignancy [10, 18, 19]. In our case we made a frozen section to distinguish if the tumor is originated from parotid tissue or the facial nerve itself. The result was reported as “mesenchymal tumor.” Because the tumor could not be separated from the nerve we had to sacrifice the temporofacial branch to resect the tumor. Benign FNS grow slowly and resection is not always indicated [20]. Several authors have reported the occurrence of facial palsy when an intraparotid facial nerve tumor was simply biopsied or carefully resected with apparent preservation of the facial nerve [21, 22], but other authors advocate its removal if it can be dissected easily off the nerve [23, 24].

Marchioni et al. suggested a classification of intraparotid FNS according to its position in the facial nerve pathway. In this classification, there are 4 types of intraparotid FNS. Type A tumors can be resected without sacrificing the facial nerve. Type B tumors can be resected with partial sacrifice peripheral branches of facial nerve or their distal divisions. Type C tumors require sacrificing the main trunk of the facial nerve for their resection, while type D tumors require sacrificing the trunk and its main divisions to be resected [24]. Our presented case was considered as type B.

As a result FNS are quite rare tumors and the preoperative diagnostic tests usually provide no valuable information. Frozen section examination that has been performed intraoperatively is recommended for both diagnosis and exclusion of malignancy. During management it seems important to behave conservatively because tumor rarely affects the facial nerve function and grows very slowly. Complete excision leads to complete cure.
